# Antibiotic Effect on Clinical Response and Remission in Pediatric Inflammatory Bowel Disease

**DOI:** 10.3390/pediatric17040077

**Published:** 2025-07-21

**Authors:** Caeley Dye, Caroline M. Sierra, Khaled Bahjri, Mallory Cohen, Gautam Nagendra

**Affiliations:** 1 Loma Linda University School of Pharmacy, Loma Linda University Health, Loma Linda, CA 92350, USA; 2 Department of Pediatric Gastroenterology, Loma Linda University Children’s Hospital, Loma Linda University Health, Loma Linda, CA 92350, USA

**Keywords:** pediatrics, inflammatory bowel disease, ulcerative colitis, Crohn’s disease, antibiotics

## Abstract

**Objective**: Gut dysbiosis has been implicated in the pathology of inflammatory bowel disease (IBD). There is some evidence to suggest that the use of antibiotic treatment can incite an early clinical response or remission when used in conjunction with standard-of-care (SOC) therapy to treat IBD-related flares. Furthermore, antibiotics have been historically investigated for use as a bridge when initiating biologic therapy while waiting for peak biologic treatment effect to occur. This study investigated and compared the time to clinical response when treated with combination antibiotics, metronidazole monotherapy, or SOC therapy in pediatric patients with an active IBD flare. **Methods**: This study was a retrospective, Institution Review Board-approved, single-centered cohort study which included patients who were less than 18 years of age with a confirmed diagnosis of IBD who received conventional treatment alone or with either combination antibiotic therapy or metronidazole monotherapy to treat an active IBD flare between March 2013 and January 2024. Patients were excluded if they received antibiotic therapy to treat an active infection, had positive stool cultures or enteric pathogen polymerase chain reaction panel, or had colonic disease limited to the rectum. **Results**: Fifty-nine patients were included and divided into metronidazole monotherapy (*n* = 18), SOC therapy (*n* = 20), and combination antibiotics (*n* = 21). The primary outcome of days to clinical response was not significantly different across all groups; however, patients who received combination antibiotics achieved the fastest time to clinical response (median (IRQ))—4 days (1, 65), compared to 7.5 days (1, 119) for the SOC group and 9 days (2, 217) for the metronidazole group. Secondary outcomes of achievement of clinical response, remission, or failure were determined to be non-significant between all groups. **Conclusions**: There is no significant difference in time to clinical response, attaining clinical response or remission, or treatment failure rate for patients treated with combination antibiotics, metronidazole monotherapy, or SOC. However, results of this study suggest that the use of combination antibiotics plus SOC may lead to a faster time to clinical response and remission compared to SOC therapy alone. Further studies are warranted to elucidate the role of antimicrobial therapy in management of pediatric IBD.

## 1. Introduction

Gut dysbiosis has been implicated in the pathogenesis of inflammatory bowel disease (IBD) and may incite a strong inflammatory host-response, contributing to the relapsing nature of IBD flares [1,2,3,4]. The cause of dysbiosis and exaggerated patient immune response is likely multifactorial and both genetic predisposition and environmental factors can increase risk [5]. As a result, there is growing interest in the utilization of antibiotics for the induction of remission by resetting colonic flora in patients with IBD [6].

Several studies have explored the use of antibiotics in adult patients with ulcerative colitis (UC) or Crohn’s disease (CD) with mixed results [2,7,8,9,10,11,12,13]. Chapman et al. found that intravenous metronidazole plus intravenous corticosteroids did not meaningfully impact time to remission in patients with acute, severe UC [14]. In 2014, Kato et al. showed that 14 days of a combination of amoxicillin, tetracycline, and metronidazole in patients with steroid-dependent UC resulted in more than half of patients experiencing a clinical response [9].

In pediatric patients, the combination of azithromycin and metronidazole compared to metronidazole monotherapy led to a significantly increased rate of remission in patients with mild-to-moderate CD [11]. In pediatric patients with acute severe colitis, Turner et al. demonstrated that a 3-week course of oral vancomycin, amoxicillin, metronidazole, and doxycycline with intravenous corticosteroids resulted in a significantly lower mean day-5 PUCAI score compared to intravenous steroids alone (*p* < 0.037) [15]. Pediatric clinical practice guidelines endorse the use of antibiotic therapy in both UC and CD, though the evidence is often low-quality and antibiotic therapy is recommended only in select patient populations [16,17,18].

The possibility that combination antibiotic therapy may induce a quicker clinical response and/or remission is especially vital in pediatric patients who experience steroid-refractory flares that oftentimes require prolonged hospital admissions [16,18]. This retrospective study evaluated the time to response, remission, and treatment failure for pediatric patients with IBD receiving combination antibiotic therapy, metronidazole monotherapy, and standard-of-care (SOC) therapies.

## 2. Materials and Methods

### 2.1. Patient Enrollment

This Institutional Review Board-approved, retrospective cohort study was conducted among pediatric patients admitted to Loma Linda University Children’s Hospital or seen at the outpatient Loma Linda Pediatric Digestive Disease Center from March 2013 to January 2024. Patients were identified through chart review using the institution’s electronic medical record and were included if they were less than 18 years of age with a confirmed diagnosis of IBD via endoscopy and/or histopathology, treated with antibiotics for an active IBD flare, and had negative stool cultures and a negative enteric pathogen polymerase chain reaction panel at the time of treatment. Patients were also included who were treated with conventional therapy for an active IBD flare. Patients were excluded if they received antibiotic therapy to treat an active infection or had colonic disease limited to the rectum. Severe drug adverse reactions attributed to antibiotic exposure, as defined by the U.S. Food and Drug Administration (FDA), were screened for during patient chart review [19].

Data collected included baseline patient demographics, including age, sex, race, ethnicity, and IBD diagnosis; hospital length of stay (LOS); relevant laboratory biomarkers, including fecal calprotectin, hemoglobin, albumin, C-reactive protein (CRP), and erythrocyte sedimentation rate (ESR); Pediatric Ulcerative Colitis Activity Index (PUCAI) and Pediatric Crohn’s Disease Activity Index (PCDAI) scores; concomitant medications that the patient was receiving at antibiotic initiation; time from antibiotic initiation to the initiation of standard-of-care therapy; the name, dose, frequency, and duration of treatment of antibiotic(s) prescribed; reasons for suspected treatment failure based on provider documentation; and the number of patients who used either metronidazole or combination antibiotic therapy as a bridge to biologic therapy.

**Figure 1:**
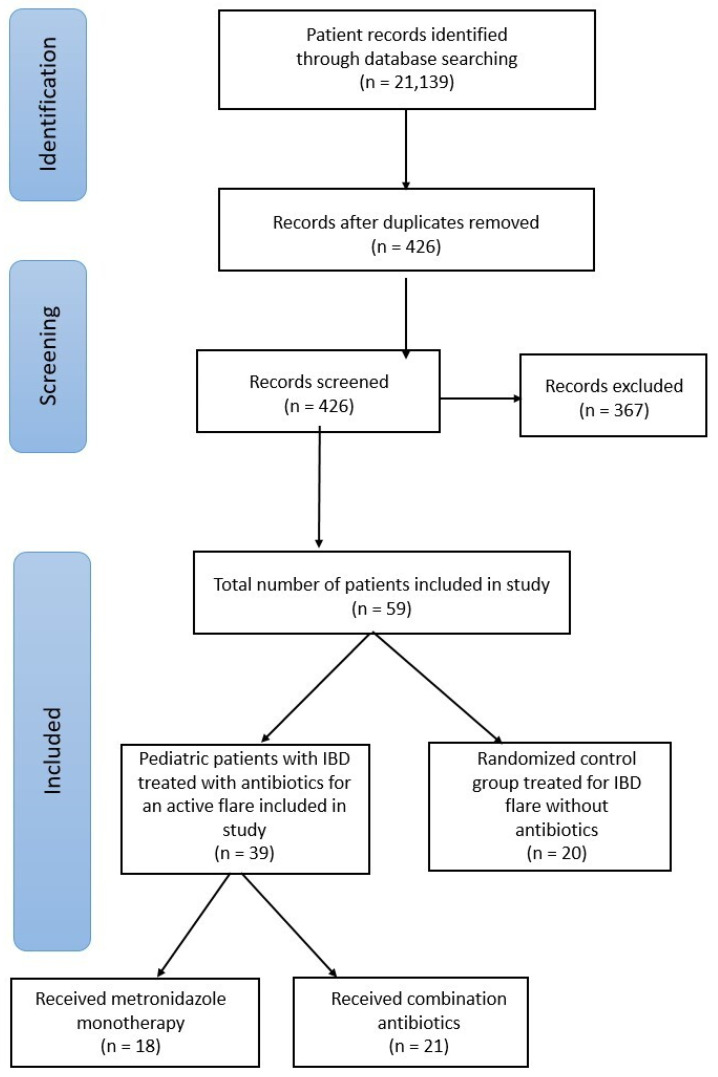
Process of patient inclusion in study.

Patients were evaluated in three cohorts: those receiving antibiotic combination therapy, metronidazole monotherapy, or SOC therapies. Patients included in the SOC group met all inclusion criteria except they did not receive antibiotic therapy for the treatment of IBD. The SOC group was formed by the random selection of patients who met the inclusion criteria but not did receive antibiotic therapy for their treatment of an IBD flare. The total number of patients in the SOC group was decided based on the number of included patients in the other treatment groups in order to maintain similar-sized cohort groups for analysis. Inclusion data and treatment groups are summarized in Figure 1.

### 2.2. Outcomes

The primary outcome was the number of days to clinical response. For patients with CD, clinical response was defined as either a decrease in PCDAI score of ≥12.5 points or a fecal calprotectin ≤ 300 μg/g. For patients with UC, clinical response was defined as either a decrease in PUCAI score of ≥20 points or a fecal calprotectin ≤ 300 μg/g. There is not a clear, linear relationship between fecal calprotectin and disease improvement/severity; however, fecal calprotectin levels < 250 μg/g have been shown to reliably indicate clinical recovery after an IBD flare and are considered an indicator for treatment success but not clinical remission [20].

Secondary outcomes included a comparison of the incidence of clinical response, clinical remission, and treatment failure after the initiation of antibiotic therapy at the following time points: 14, 28, 100, and greater than 100 days. For the purpose of this review, SOC included steroids and/or biologic therapy.

Treatment failure was defined as re-initiation of high-dose steroids, rehospitalization, emergent intestinal therapy, or all-cause mortality. Clinical remission for UC was defined as either a PUCAI score of <10 points or a fecal calprotectin ≤ 100 μg/g. Clinical remission for CD was defined as either a PCDAI score of <10 points or a fecal calprotectin ≤ 100 μg/g. Definitions of clinical response and clinical remission for UC and CD were derived from the 2018 ECCO-ESPGHAN Guideline on Management of Pediatric Ulcerative Colitis and the 2020 ECCO-ESPGHAN Guideline on Management of Pediatric Crohn’s Disease, respectively [16,17,18].

### 2.3. Statistical Analyses

Descriptive statistics were measured for each treatment group. Quantitative variables were described using median and interquartile range (IRQ) for nonparametric data or mean and standard deviation for parametric data. Qualitative variables were described using frequency and percentage. Fisher’s exact and Kruskal Wallis tests were used to assess the association of the qualitative variables and quantitative variables, respectively, among the three treatment groups. Data analysis was performed at an alpha of 0.05 using SPSS 29.0.

## 3. Results

### 3.1. Baseline Characteristics

Out of 5788 patients screened, 21 patients who were treated with combination antibiotics and 18 treated with metronidazole monotherapy met all inclusion criteria and were included for review. A total of 20 patients were derived from the remaining patients who met inclusion criteria to create the control group.

Patients were predominantly of adolescent age (13–14 years) and were either White or Hispanic males. There was an equal representation of patients with UC and CD in both the metronidazole monotherapy (9, 50%) and SOC groups (10, 50%); most patients who received combination antibiotics (16, 76%) had UC. The most common concomitant medications that patients were receiving when antibiotics were initiated were prednisone, methylprednisolone, mesalamine, infliximab, or adalimumab. See Table 1 for a complete summary of baseline characteristics.

The majority of patients who received combination antibiotics were prescribed a three-drug combination of metronidazole, amoxicillin, and doxycycline (17, 81%). Alternate antibiotic regimens included metronidazole and ciprofloxacin (2, 9%); metronidazole, amoxicillin, and sulfamethoxazole–trimethoprim (1, 5%); and ciprofloxacin monotherapy (1, 5%). Patients on metronidazole and combination antibiotics received a median duration of therapy of 11.5 days and 14 days, respectively.

### 3.2. Clinical Outcomes

Patients receiving combination antibiotics had the fastest time to clinical response with a median (IRQ) of 4 days (1, 65), followed by 7.5 days (1, 119) for the SOC group and 9 days (2, 217) for the metronidazole group. More patients who received combination antibiotics (18/21, 86%) achieved a clinical response within the first 14 days compared to the metronidazole (11/18, 60%) and SOC groups (11/18, 60%). No significant difference was detected in time to clinical response between all three treatment arms (*p* = 0.112). Outcomes of the three treatment groups are provided in Table 2.

Patients who had experienced a clinical response in all treatment arms generally achieved a response within 14 days of treatment initiation; however, there was no significant difference detected in achieving a clinical response between all three groups (*p* = 0.516). Patients who received combination antibiotics had a larger percentage of patients who experienced a clinical response within 14 days (18, 86%) compared to metronidazole alone (11, 61%) or the SOC group (11, 55%). A larger proportion of patients were treated during hospital admission compared to outpatient treatment in all three treatment arms. Out of 20 patients in the SOC group, 2 patients (10%) treated in the outpatient setting were unable to achieve a clinical response.

More patients receiving metronidazole monotherapy (16/18, 88%) and combination antibiotics (15/21, 71%) experienced clinical remission at all time points compared to patients receiving standard of care (11/20, 55%). There was no significant difference in time to clinical remission between the three treatment groups (*p* = 0.775). Patients receiving combination antibiotics (5/15, 33%) and metronidazole monotherapy (5/16, 31%) had similar rates of remission within 14 days of treatment initiation. In contrast, patients who received SOC had a lower incidence of clinical remission within 14 days (2/11, 18%). Patients in the SOC group had the highest cumulative incidence of documented treatment failure that occurred 28 days and beyond (11/20, 55%) compared to patients receiving combination antibiotics (5/21, 24%) and metronidazole monotherapy (2/18, 11%). There was no significant difference in the rate of clinical failure between all three groups (*p* = 0.460).

Combination antibiotics were utilized more frequently (*n* = 8, 38%) than metronidazole therapy (*n* = 3, 0.2%) as a bridge to biologic therapy. However, both metronidazole monotherapy and combination antibiotics were primarily used as adjunctive agents to treat UC and CD flares. On the contrary, only a minority of patients receiving metronidazole monotherapy were using steroids concomitantly. An analysis of antibiotic usage is provided in Table 3**.**

No patients who received either combination antibiotic therapy or metronidazole monotherapy experienced clinically relevant adverse reactions attributable to the use of antibiotics.

### 3.3. Changes in Clinical Scoring and Labs

Patients who received combination antibiotic therapy had the greatest observed decrease in average absolute fecal calprotectin levels before and after treatment completion (2149 mcg/g vs. 1442 mcg/g). Patients in the control group were also observed to have had a smaller decrease in average absolute fecal calprotectin levels after treatment (1070 mcg/g vs. 759 mcg/g). Contrarily, patients who received metronidazole monotherapy were observed to have increased average absolute fecal calprotectin levels after treatment (834 mcg/g vs. 1066 mcg/g). Patients with UC receiving SOC therapy had the largest observed decrease in PUCAI scores after treatment, with a difference of 41 points, followed by combination antibiotics (32 points) and metronidazole monotherapy (7 points). Other clinical data can be found in Table 4.

## 4. Discussion

The results of this study suggest that use of combination antibiotics may lead to a faster time to clinical response and clinical remission compared to standard-of-care therapy in pediatric patients with IBD. The majority of patients who received combination antibiotics were prescribed a regimen consisting of metronidazole, amoxicillin, and doxycycline. However, promising, the exact antibiotic regimen that can provide the most benefit to this patient population has yet to be elucidated. The results of this study contribute to the growing evidence that antibiotics may play a larger role than what is recommended in current clinical practice guidelines. This may be especially true in pediatric patients admitted to the hospital for IBD flares refractory to first-line treatment, as the majority of patients in this study receiving combination antibiotics were simultaneously receiving corticosteroid therapy. Although not statistically significant, our study observed that the majority of patients receiving either metronidazole monotherapy or combination antibiotics experienced a clinical response within the first two weeks of therapy.

In a retrospective cohort study, Breton et al. evaluated the rate of clinical response and remission after 3 ± 1 weeks of combination antibiotics amoxicillin, metronidazole, and doxycycline in a pediatric population where 80% of patients were steroid-dependent [7]. Though the authors used a different antibiotic regimen than the present study, they showed a significant improvement in disease activity—63.5% of patients experienced a clinical response and 39.7% of patients entered clinical remission. Five additional patients entered clinical remission after week 3 while using combination antibiotic therapy as bridge therapy [7]. Turner et al. studied 15 children with moderate to severe UC who were treatment-refractory (corticosteroid resistant or refractory to multiple immunosuppressants) and received triple antibiotic therapy with amoxicillin, metronidazole, and doxycycline [15]. Nine patients (60%) achieved complete remission—two patients achieved remission after one week, six patients after two weeks, and one patient after three weeks [15]. Our study provides greater evidence that the target population for antibiotic therapy may be refractory cases, with optimistic results paralleling previous studies [7,15].

In practice, patients who are initiated on or transitioning to TNF-alpha inhibitor therapy, commonly infliximab or adalimumab, may have drastically different clinical response times dependent on factors including drug dosing, age at initiation, disease state, and history of surgery [20]. Infliximab’s general time to response ranges from 2 to 8 weeks, while adalimumab ranges from 4–8 weeks [20]. In this study, the majority of patients who received a 14-day treatment of combination antibiotics as bridge therapy have ulcerative colitis and were hospitalized secondary to steroid-refractory flares. Evidence for their use in a similar patient population with positive treatment outcomes was shown in the results of the Breton et al. study, which observed a significant clinical remission rate at week 3 after the initiation of combination antibiotics when used as a bridge for treatment-refractory patients being transitioned to either vedolizumab or ustekinumab [7].

Antibiotic therapies for inducing remission in IBD vary in the literature, though all target pathogens associated with inflammation and disease flares. One of the earliest studies that identified causative bacteria in patients with UC showed that *Fusobacterium varium* was correlated to the development of inflamed ulcerative lesions [21]. More recent gut microbiome studies in pediatric patients demonstrated a disproportionate presence of oral flora in patients with IBD, particularly *Veillonella*, *Haemophilus*, and *Eikenella* spp. [1]. Other pathogenic species that have been seen growing in excess are *Yersinia enterocolitica*, *Listeria monocytogenes*, *Mycobacterium avium*, and *Escherichia coli* [22]. The addition of azithromycin has been identified as promising due to its ability to penetrate multiple intestinal compartments, including the intestinal lumen, biofilms in the mucus layer which may consist of adherent and invasive *Escherichia coli*, and macrophages [6,23,24,25]. The combination of antibiotics used in the present study provides adequate coverage for both the Gram-negative and anaerobic pathogens that are correlated to precipitating IBD flares.

Use of antibiotics without infectious pathology should be weighed against possible risks, including the instigation of bacterial resistance and impact on the diversity of bacterial species in the intestinal microbiome [6,25]. Turner et al. observed increased resistance against ciprofloxacin, doxycycline, and tetracycline by day 30 in patients receiving antibiotics but not in those receiving steroid alone; resistance levels for ciprofloxacin and tetracycline returned to baseline 90 days post-antibiotic treatment. Sprockett et al. found, in treatment groups modeled after Turner and colleagues’ study, that patients receiving metronidazole monotherapy and combination metronidazole and azithromycin showed a significant decrease in alpha diversity in gut microbiota after four weeks of antibiotic treatment. A rebound in gut diversity was seen in both treatment groups after 12 weeks, and the metronidazole group had recovered diversity comparable to baseline [25]. To limit antibiotic exposure and minimize patient risk, it may be advisable to discontinue combination antibiotics if no treatment response is seen within five days [26].

A significant limitation to this study was missing data from patients’ medical records. The lack of clinical severity score reporting may have led to unaccounted clinical response or remission. The decision to calculate a PCDAI or PUCAI score and the frequency of lab draws throughout a patient’s hospital admission are not protocolized and were inconsistently documented. Only lab values and PUCAI scores that were reported before and after the completion of treatment in the electronic medical record were reported in this study. Furthermore, the lack of existing patient data compromised the ability to match information between cohorts and was therefore not performed. It may be beneficial for future studies to protocolize patient assessment to better understand how these treatments affect patients in both the short and long term. Another limitation to the study was a small sample size. While a trend favoring combination antibiotics usage was noted, the treatment effect may have been hidden due to the sample size.

The results of this study suggest that combination antibiotic therapy can serve as a bridge to biologic therapy, improving time to clinical response and remission over standard-of-care therapy. More patients in the combination antibiotics group achieved clinical response within 14 days and on average had the quickest time to clinical response. The thought of incorporating adjunctive antibiotics appears valid in the setting of the overarching theory that gut dysbiosis might play a causative role in the pathogenesis of IBD. However, there is limited evidence to support the routine use of antibiotics for this indication at this time. There is a need for more studies to investigate the effects of antibiotic therapy in UC compared to CD. More robust studies, including randomized controlled trials, are needed to elucidate the clinical impact of antimicrobial agents in the management of pediatric IBD.

## Figures and Tables

**Table 1:** Baseline characteristics.

Baseline Characteristic	Metronidazole (*n* = 18)	Combination Antibiotics (*n* = 21)	Control Group (*n* = 20)
Age (years), mean ± SD ^1^	13 ± 3.9	14 ± 2.56	13 ± 3
Sex (male), n (%)	9 (50)	10 (47)	16 (80)
Weight (kg), median (min, max)	109 (12, 120)	52 (24, 75)	41 (21, 112)
Race, n (%)			
White	15 (83)	15 (71)	18 (90)
Asian	1 (6)	1 (5)	1 (5)
Not recorded	2 (11)	5 (24)	1 (5)
Ethnicity, n (%)			
Hispanic or Latino	9 (50)	12 (57)	10 (50)
Non-Hispanic	9 (50)	9 (43)	10 (50)
Type of IBD, n (%)			
Ulcerative Colitis	9 (50)	16 (76)	10 (50)
Crohn’s Disease	9 (50)	5 (24)	10 (50)
Maintenance Therapy, n (%)			
Adalimumab	3 (17)	5 (24)	4 (20)
Azathioprine	1 (6)	0	2 (10)
Budesonide	0	1 (5)	1 (5)
Hydrocortisone	4 (22)	2 (10)	0
Infliximab	4 (22)	7 (33)	7 (35)
Mesalamine	8 (44)	7 (33)	7 (35)
Methotrexate	2 (11)	1 (5)	0
Methylprednisolone	4 (22)	5 (24)	4 (20)
Prednisone	7 (39)	12 (57)	9 (45)
Sulfasalazine	2 (11)	1 (5)	2 (10)
Ustekinumab	1 (6)	1 (5)	3 (15)
Vedolizumab	0	1 (5)	1 (5)
6-MMP	1 (6)	1 (5)	0

^1^ SD—standard deviation; IBD—inflammatory bowel disease; 6-MMP—6-methylmercaptopurine.

**Table 2:** Clinical outcomes.

Primary Outcome					
	Metronidazole (*n* = 18)	Control Group (*n* = 20)	Combination Antibiotics (*n* = 21)		*p*-Value
Time to clinical response, median (min, max)	9 (2, 217)	7.5 (1, 119)	4 (1, 65)		0.112
**Secondary Outcome**					
	Time (days)	Metronidazole	Control Group	Combination Antibiotics	*p*-value
Treatment failure, n (%)	No Treatment Failure	16 (89)	9 (45)	16 (76)	0.460
	1 through 14	0	0	0	
	15 through 28	0	2 (10)	0	
	29 through 100	1 (6)	7 (35)	2 (10)	
	>100	1 (6)	2 (10)	3 (14)	
Clinical remission, n (%)	1 through 14	5 (28)	2 (10)	5 (24)	0.775
	15 through 28	0 (0)	0	1 (5)	
	29 through 100	7 (39)	6 (30)	7 (33)	
	>100	4 (22)	3 (15)	2 (10)	
Clinical response, n (%)	1 through 14	11 (61)	11 (55)	18 (86)	0.516
	15 through 28	2 (11)	3 (15)	2 (10)	
	29 through 100	3 (17)	2 (10)	1 (5)	
	>100	2 (11)	2 (10)	0	

**Table 3:** Antibiotic usage.

Antibiotic Usage	Metronidazole (*n* = 18)	Combination Antibiotics (*n*= 21)
No bridge therapy, n (%)	15 (83)	13 (62)
Inpatient, n (%)	8 (53)	9 (69)
Outpatient, n (%)	7 (46)	4 (31)
Bridge therapy, n (%)	3 (0.2)	8 (38)
Inpatient, n (%)	3 (100)	7 (88)
Outpatient, n (%)	0	1 (12)
Antibiotic regimens		
Metronidazole, Amoxicillin, Doxycycline	-	17 (81)
Metronidazole, Ciprofloxacin	-	2 (9)
Metronidazole, Amoxicillin,	-	1 (5)
Sulfamethoxazole–Trimethoprim		
Ciprofloxacin monotherapy	-	1 (5)
Antibiotic duration (days), median (min, max)	11.5 (4, 94)	14 (1, 124)
Patients receiving steroids on antibiotics, n	4	15
Days patient on steroids prior to antibiotic initiation, median (min, max)	4 (0, 25)	6 (0, 16)
Days from initiation of antibiotics to initiation of anti-TNF inhibitor	17 (13, 19)	3.5 (0, 17)

**Table 4:** Change in laboratory biomarkers and clinical scoring.

Lab	Timeframe ^1^	Metronidazole	Control Group	Combination Antibiotics
Hemoglobin (g/dL)	Before Starting Treatment	11 ± 2	11 ± 3	10 (5, 14)
	After Completing Treatment	11 ± 3	13 (7, 16)	10 (8,12)
Albumin (g/dL)	Before Starting Treatment	4 ± 0.4	4 (1, 5)	3 ± 1
	After Completing Treatment	4 ± 1	4 ± 1	4 ± 1
C-reactive Protein (mg/dL)	Before Starting Treatment	0.9 (0.3, 21.3)	1.4 (0.3, 34.8)	5 (0.3, 15)
	After Completing Treatment	1 ± 1	1.2 (0.1, 17)	5 (0.3, 21)
Erythrocyte Sedimentation Rate (mm/h)	Before Starting Treatment	29 ± 21	37 ± 31	40 (2, 97)
	After Completing Treatment	17 ± 12	19 ± 18	40 (11, 87)
Absolute Fecal Calprotectin (mcg/g)	Before Starting Treatment	834 ± 499	1070 ± 1112	2149 ± 1011
	After Completing Treatment	1066 ± 579	759 ± 1126	1442 ± 1045
PUCAI ^2^ Score	Before Starting Treatment	45 ± 40	56 ± 6	55 (35, 85)
	After Completing Treatment	38 ± 33	15 ± 4	23 ± 21

^1^ Results expressed either as mean ± standard deviation for parametric data or median (min, max) for non-parametric data ^2^ Pediatric Ulcerative Colitis Activity Index.

## Data Availability

The data presented in this study are available on request from the corresponding author to protect patient confidentiality.

## Abbreviations

The following abbreviations are used in this manuscript

CD	Crohn’s disease
CRP	C-reactive protein
FDA	U.S. Food and Drug Administration
IBD	Inflammatory bowel disease
LOS	Length of stay
PCDAI	Pediatric Crohn’s Disease Activity Index
PUCAI	Pediatric Ulcerative Colitis Activity Index
SOC	Standard-of-care
UC	Ulcerative colitis
